# Perceived parenting and adolescents’ adjustment

**DOI:** 10.1186/s41155-018-0088-x

**Published:** 2018-03-07

**Authors:** Joana Jaureguizar, Elena Bernaras, Paola Bully, Maite Garaigordobil

**Affiliations:** 1 Department of Developmental and Educational Psychology, Faculty of Education of Bilbao, University of the Basque Country (Spain), Barrio Sarriena s/n, 48940 Leioa, Spain; 2 Department of Developmental and Educational Psychology, Faculty of Education, Philosophy and Anthropology (Donostia–San Sebastián), University of the Basque Country (Spain), Plaza Oñati 3, 20018 Donostia–San Sebastián, Spain; 3 Research Unit of Primary Attention (Bizkaia), Basque Health Service-Osakidetza, C/ Luis Power, 18, 4, 48014 Bilbao, Spain; 4 Department of Personality, Assessment and Psychological Treatments, Faculty of Psychology, University of the Basque Country, Avda Tolosa 70, 20018 Donostia–San Sebastian, Spain

**Keywords:** Adolescence, Parenting, Adjustment, Control, Warmth

## Abstract

Adolescence is an important developmental period that is characterised by heightened problems of adjustment. The aim of this study is to analyse adolescents’ adjustment, and to explore the typologies and dimensions of parenting, and thus to determine the relationships between these factors. The sample comprised 1285 adolescent students aged 12 to 16 from the Basque Country (Spain). The students filled out the self-report of the Behavior Assessment System for Children (BASC) and the Parental Acceptance-Rejection/Control Questionnaire, (PARQ/Control). Differences by age were found in the adolescents’ school maladjustment and parenting style perception. Moreover, perceptions of little parental warmth were related to higher levels of clinical and school maladjustment, and the lower the parental control, the greater the clinical maladjustment. Finally, the results obtained revealed that the interaction between the mothers’ and fathers’ parenting styles was significant only for clinical maladjustment; those students with neglectful mothers and authoritative fathers presented the highest level of clinical maladjustment, followed by other combinations of neglectful mothers. Furthermore, the students from neglectful and authoritarian families presented the highest levels of school maladjustment, without differences between neglectful and authoritarian or between indulgent and authoritative families.

## Background

Adolescence is a developmental period between childhood and adulthood that is characterised by many physical, psychological and social changes that require adaptation. This can be an opportunity to enhance development, but it can also highlight the vulnerabilities of the adolescent. Although most young people cope adequately with such changes, this developmental period is characterised by an increased presence of externalising and internalising problems (Donaldson, Gordon, Melvin, Barton, & Fitzgerald, 2014; Merikangas et al., 2010).

Analysis of differences in adolescents’ adjustment by sex reveals a tendency for boys to be less well adjusted to school, with more behavioural problems, more negative attitudes toward teachers and lower levels of achievement (Holden, 2002; Jaureguizar, Ibabe, & Strauss, 2013; Lam et al., 2012). Girls, on the other hand, are less emotionally adjusted and present more depression and anxiety (Thapar, Collishaw, Pine, & Thapar, 2012; Waite & Creswell, 2014). Moreover, in respect of the age, increasing age has been associated with higher prevalence of depression and anxiety (Zubrick et al., 2017), while externalising problems are generally stable over the course of development (Li, Johnson, Musci, & Riley, 2017).

Taking into account that adjustment problems in childhood and adolescence might be related to psychiatric disorders in adulthood, many studies have emphasised the need to identify factors related to such adjustment problems (Fuentes, García, Gracia, & Lila, 2011; Gerard & Booth, 2015; Jaureguizar, Bernaras, Ibabe, & Sarasa, 2012; Waite & Creswell, 2014).

Although peers play a major role in socialisation during adolescence, the family is still an important source of support, reference and education. Moreover, parenting has long been identified as one of the most important influential factors in children’s and adolescents’ psychosocial adjustment (Lengua & Kovacs, 2005; Rodríguez-Fernandez, Droguet, & Revuelta, 2012). Studies of parenting can be divided into two main perspectives: research focused on dimensions of parenting and research focused on typologies (Darling & Steinberg, 1993; O’Connor, 2002).

Two main dimensions of parenting behaviour have been studied: warmth and control. Warmth is defined as involvement and the expression of positive affect, regard and concern by parents and/or other primary caregivers (Rohner & Khaleque, 2005). Studies have identified a significant link between parental warmth and the positive adjustment of adolescents (Barber, Stolz, & Olsen, 2005; Heider, Matschinger, Bernet, Alonso, & Angermeyer, 2006). In this respect, Rohner and Khaleque (2005) reported that up to 26% of the variability of children’s psychological adjustment could be accounted for by their perceptions of acceptance or rejection by the primary caregivers. By contrast, clinical and community studies have found that lower parental support is related to greater depression and anxiety among adolescents (Johnson & Greenberg, 2013; Sheeber, Davis, Leve, Hops, & Tildesley, 2007; Yap, Pilkington, Ryan, & Jorm, 2014), lower academic performance (Gerard & Booth, 2015; Ratelle, Larose, Guay, & Senecal, 2005), weaker self-esteem (Fuentes et al., 2011) and behavioural problems such as delinquency (Heaven, Newbury, & Mak, 2004) or substance use (Calafat, García, Juan, Becoña, & Fernández-Hermida, 2014).

Research findings on the influence of parental control on adolescents’ adjustment have been less consistent (Heider et al., 2006; Kerr & Stattin, 2000), probably due to the use of different conceptualisations of this construct, i.e. whether it is understood as behavioural or as psychological control. Behavioural control by parents is viewed as the extent to which they regulate or manage their children’s behaviour, for instance by insisting on compliance with rules, demands or directives (Rohner & Khaleque, 2003). Several studies have observed positive relationships between parental control and adolescents’ adjustment, due to the fact that such control implies knowledge of the child’s whereabouts, activities and companions (Jacobson & Crockett, 2000; Kerr & Stattin, 2000). However, when behavioural control becomes over-strict, the consequences for adolescents’ emotional adjustment could be negative (Maccoby & Martin, 1983). The second conceptualisation, that of psychological control, describes intrusive parenting tactics (for example, guilt-induction or shaming) (Barber, 1996) that keep adolescents emotionally dependent on their parents, thus controlling behaviour and limiting the development of autonomy (Reitz, Decovic, & Meijer, 2006) and adjustment across cultures (Soenens, Park, Vansteenkiste, & Mouratidis, 2012).

Studies of parenting typologies have traditionally adopted a two-dimensional framework (warmth or acceptance vs. control or strictness) with four types of parental socialisation style: authoritative (high in warmth and control), authoritarian (high in control but low in warmth), indulgent (high in warmth but low in control) and neglectful (low in warmth and in control) (Baumrind, 1991; Maccoby & Martin, 1983; Steinberg, Blatt-Eisengart, & Cauffman, 2006).

Using this distinction between styles of socialisation, various studies have analysed the relationship between these styles and children’s adjustment/maladjustment coming to different conclusions. The neglectful style has been associated with problems of social adjustment (such as impulsivity, substance abuse, crime or child-to-parent violence) and of personal adjustment (such as low self-esteem, anxiety or depression) (Gámez-Guadix, Jaureguizar, Almendros, & Carrobles, 2012; Milevsky, Schlechter, Netter, & Keehn, 2007; Oliva, Parra, & Arranz, 2008; Steinberg, 2001). The authoritarian style, on the other hand, has been linked to emotional maladjustment (in the form of depression, low self-esteem or low self-confidence) (Garber, Robinson, & Valentiner, 1997). The indulgent style is associated with psycho-social problems (substance abuse, antisocial behaviour, etc.) (Adalbjarnardottir & Hafsteinsson, 2001; Steinberg et al., 2006), and the authoritative style with higher levels of psycho-social adjustment (better self-esteem, good adjustment to school, satisfactory academic performance, psychological maturity and fewer behavioural problems) (Baumrind, 1997; Im-Bolter, Zadeh, & Ling, 2013; Kritzas & Grobler, 2005; Pelegrina, García, & Casanova, 2002). However, many studies claim that equivalent outcomes are achieved in indulgent and authoritative families, and even that children raised in indulgent families may present higher levels of adjustment (Calafat et al., 2014; Esteve, 2005; Gámez-Guadix et al., 2012; García & Gracia, 2009, 2010; López-Romero, Romero, & Villar, 2012). According to Musitu and García (2001), indulgent parents interact with their children as if they were mature and capable of self-control, consult with them about important household decisions and avoid the use of imposition and coercive measures of control.

A final issue that should be taken into account in considering the literature about relationships between parenting and adolescents’ adjustment is that of the sex of the parents. The vast majority of studies on childhood and adolescent adjustment have focused on mother-children relationships, or on joint parenting. However, a few studies have considered these issues controlling by sex and have observed different effects of maternal and paternal parenting on children’s adjustment (Hoeve, Dubas, Eichelsheim, van der Laan, Smeenk, et al., 2009; McKinney, Donnelly, & Renk, 2008), although other studies have not found strong differences in this respect (Sheeber et al., 2007).

Taking into account previous work in this field, and given the lack of a single conclusion, the aim of our study is to analyse adolescents’ adjustment, and to explore the typologies and dimensions of parenting, and thus to determine the relationships between these factors. The following hypotheses are proposed:

- Hypothesis 1: Maladjustment is more likely to occur when adolescents perceive low levels of warmth and acceptance from their parents.
- Hypothesis 2: Maladjustment is more likely to occur when adolescents perceive low levels of control and supervision from their parents.
- Hypothesis 3: Maladjustment is more likely to occur when adolescents perceive an authoritarian parenting style.

## Methods

### Sample and procedure

The sample was obtained through convenience sampling (Coolican, 1994). In order to obtain a heterogeneous sample, we contacted both public and grant-assisted private schools and also made sure to include schools not only from the provincial capitals, but also from small towns and villages in the three provinces of the Basque Country (Spain). The sample consisted of 1285 adolescent students aged 12 to 16 years: 636 were girls (mean age = 13.73; SD = 1.59) and 649 were boys (mean age = 13.72; SD = 1.28).

Before contacting the schools, the procedure was reviewed by the Ethics Committee for Research with Human Beings at the University of the Basque Country, which gave its approval for the study to be carried out.

In face-to-face interviews with head teachers, we explained the aims of the study, the procedures to be followed and the instrument to be used. Once a school had agreed to participate, the conditions were set for the students’ families to receive the informed consent documents. Those families that allowed their children to take part returned the informed consent forms to the school, and we then proceeded to apply the instruments, which were applied in group format by members of the research team. The instructions for filling out the questionnaires were read aloud in the classroom and the students filled out the questionnaires in normal lesson time.

### Measurement

#### Behavior assessment system for children and adolescents (BASC) (Reynolds & Kamphaus, 1992, Spanish adaptation by González, Fernández, Pérez, & Santamaría, 2004, in its self-report version for adolescents aged 12 to 18 (S3)

The S3 personality self-report is an inventory composed of 185 statements, to which respondents are required to answer ‘True’ or ‘False’ (for example, ‘I hate school’; ‘No one understands me’). Four global dimensions are obtained from analysis of the replies made: personal adjustment, clinical maladjustment, school maladjustment and index of emotional symptoms. In the present study, only the dimensions of clinical and school maladjustment were explored. Clinical maladjustment is characterised as a general index of the distress reflected in clinical disorders and problems of internalisation. School maladjustment, on the other hand, is a measure of generalised maladjustment in this environment (negative attitudes toward school personnel and the structure of the educational process in general), which is often accompanied by academic difficulties. Three categorizations are obtained from the results: no maladjustment (*T*_score_ < 60), at risk of maladjustment (*T*_score_ 60–69) and clinically significant maladjustment (*T*_score_ > 69). The Cronbach’s alpha scores for these two dimensions were satisfactory, at 0.94 for clinical maladjustment and 0.83 for school maladjustment.

#### Parental acceptance-rejection/control questionnaire (PARQ/Control)-Spanish version (Rohner, 1990)

Two scales of this questionnaire were used: the *Warmth/Affection Scale* (Rohner, Saavedra, & Granum, 1978) and the *Parental Control Scale* (Rohner, 1989; Rohner & Khaleque, 2003). The PARQ/Control, with its warmth/affection and control scales, assesses individual perceptions of warmth and parental control among children aged 7 years and over and adolescents. The warmth/affection and control scales consist of 20 and 13 items, respectively, to be assessed on a Likert scale of four possible answers: “Almost always true”, “Sometimes true”, “Rarely true”, “Almost never true”. Each item must be answered separately for the maternal and paternal relationships, to differentiate the results obtained for the mother and father (or guardians). The warmth/affection scale evaluates adolescents’ perceptions of the love and affection received from their parents (or guardians): acceptance of their personality, interest in their activities and well-being, enjoying free time together, ability to comfort, console, praise, embrace or express their love through words and actions. The parental control scale, on the other hand, evaluates adolescents’ perceptions of the behavioural control (permissiveness or strict control) exercised by their parents or guardians. The vast majority of the questionnaire items refer to the behaviour of parents, rather than their attitudes, to avoid possible difficulties in accounting for the relationship between attitudes and behaviour. The Cronbach’s alpha scores for these two scales were satisfactory, at 0.94 for warmth/affection and 0.86 for parental control.

To obtain the four categories of parenting styles, the mothers’ and fathers’ scores for warmth and control were dichotomised (high vs. low) by the median (García & Gracia, 2010; Lamborn, Mounts, Steinberg, & Dornbusch, 1991), such that parent’s scoring low in warmth and control were classified as neglectful; low in warmth and high in control as authoritarian; high in warmth and low in control as indulgent; and high in warmth and control as authoritative.

### Data analysis

First, the distributions of the severity levels and scores associated with clinical and school maladjustment were described using frequencies, means and standard deviations. To test differences by gender (male vs. female) and age (12-year-olds; 13- to14-year-olds; and 15- to 16-year-olds) contingency tables, chi-square (*χ*^2^) and Cramer’s *V* (*V*_Cramer_) as a measure of effect size in categorical variables and analysis of variance (ANOVA), post hoc tests of between-group comparisons (Tukey’s HSD; *HSD*) and Hedges’ *g* (*g´*) in quantitative variables were used. The same procedure was carried out to the analysis for the dimensions of warmth and control and parenting styles.

Next, a successive multivariate analysis of variance (MANOVA) was performed to test differences between levels of clinical and school maladjustment among students with different levels of parental warmth and control and for authoritative, authoritarian, neglectful or indulgent parents. In light of the significant relation between adolescent age and parenting style, analyses were also conducted both with, and without, age as covariate; because the results did not change when this covariate was included, only findings from analyses that age did not covariate are reported. Follow-up univariate *F* tests were performed within the variables that presented significant overall multivariate differences, and significant results in the univariate tests were followed up with Tukey’s HSD comparisons with Bonferroni’s correction between all possible pairs of means.

## Results

### Clinical and school maladjustment

As severity levels of maladjustment is concerned, the BASC-S3 results indicated that 5% (*n* = 59) of the sample reported clinically significant clinical maladjustment, and a further 10.5% (*n* = 124) were at risk of clinical maladjustment. 6.6% (*n* = 78) of the sample reported clinically significant school maladjustment, and a further 18.8% (*n* = 221) were considered to be at risk. Severity of maladjustment did not vary as a function of adolescents’ sex [clinical: *χ*^2^ (2) = 3.32, *p* = .19, *V*_Cramer_ = .05; school: *χ*^2^ (2) = 3.34, *p* = .18, *V*_Cramer_ = .05] but did vary as a function of adolescents’ age: in the younger adolescents group, there was a higher percentage than expected by chance who were in clinically significant clinical maladjustment category [*χ*^2^ (4) = 11.45, *p* = .02, *V*_Cramer_ = .07] and in the middle adolescents group, the clinically significant school maladjustment was more prevalent than expected [*χ*^2^ (4) = 17.65, *p* < .01, *V*_Cramer_ = .09] (see Table 1).

**Table 1:** Relation between clinical and school maladjustment and age group

		Clinical maladjustment				School maladjustment			
		Age group			Total	Age group			Total
		12 years	13–14 years	15–16 years		12 years	13–14 years	15–16 years	
	No								
	*n*	214	440	346	1000	214	365	299	878
	%	82.6	83.8	86.7	84.5	82.6	69.8	75.7	74.6
	CR	− 1.0	− 0.6	1.5		3.4	− 3.4	0.6	
	Risk								
	*n*	24	66	34	124	33	112	76	221
	%	9.3	12.6	8.5	10.5	12.7	21.4	19.2	18.8
	CR	− 0.7	2.1	− 1.6		− 2.8	2.1	0.3	
	Clinically significant								
	*n*	21	19	19	59	12	46	20	78
	%	8.1	3.6	4.8	5.0	4.6	8.8	5.1	6.6
	CR	2.6	− 1.9	− 0.3		− 1.5	2.7	− 1.5	

*CR* corrected residuals

**Fig. 1:**
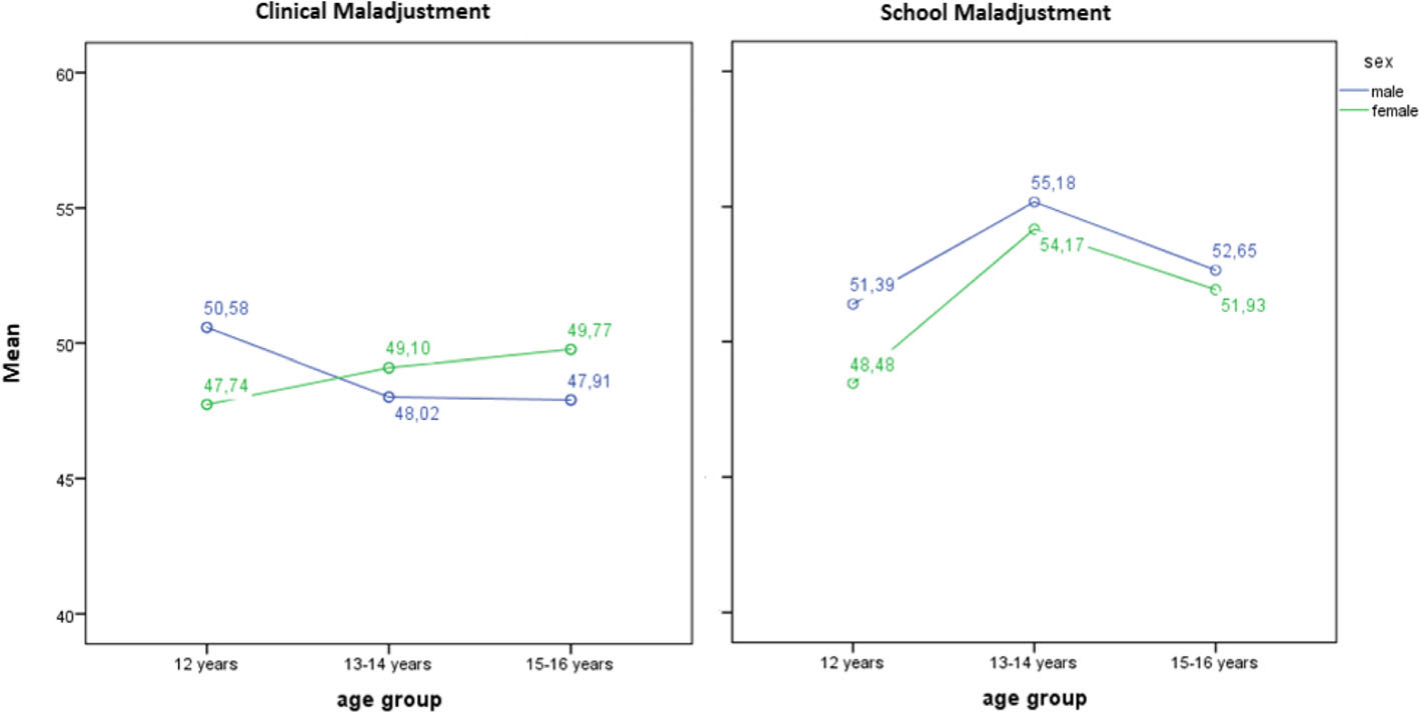
Adolescents’ clinical and school maladjustment by sex and age

As regards clinical maladjustment, Fig. 1 shows that as girls become older, they score higher in clinical maladjustment, but that boys present the opposite trend. The results of the 2 × 3 ANOVA revealed no statistically significant main effects of sex [*F* (1, 1177) = 0.003, *p* = .96] or age [*F* (2, 1177) = 0.26, *p* = .77], but the interaction between the two variables was significant *[F* (2, 1177) = 3.81, *p* = .02]. However, the differences between the six groups generated from the interaction between sex and age were so small that they were not statistically significant.

The same procedure was followed for school maladjustment. Sex, but with low effect size, [*F* (1, 1171) = 6.58, *p* = .01, *M*_males_ = 53.40, SD_males_ = 10.26, *M*_females_ = 52.32, SD_females_ = 9.82, *g´* = 0.11] and age [*F* (2, 1171) = 20.51, *p* < .001, *M*_12 years_ = 50.14, SD_12 years_ = 9.87, *M*_13–14 years_ = 54.65, SD_13–14 years_ = 9.80, *M*_15–16 years_ = 52.30, SD_15–16 years_ = 10.09, *g´*_12–14 years_ = − 0.46, *g´*_12–16 years_ = 0.21, *g´*_13–16 years_ = − 0.24] were statistically significant. Their interaction variables were not significant [*F* (2, 1171) = 1.07, *p* = .34]. The 13–14 age group presented the highest levels of school maladjustment.

### Parenting dimensions and styles

Although there were no significant differences between adolescents’ perceptions of their mothers’ and fathers’ parenting styles and dimensions [warmth: *F*_Friedman_ (1) = 0.00; *p* = .999; control: *F*_Friedman_ (1) = 0.21; *p* = .647; style: *F*_Friedman_ (1) = 1.33; *p* = .251], Table 2 shows the results for the parenting styles and dimensions of fathers and mothers, separately, as reported by the students in our study sample.

**Table 2:** Descriptive results for fathers’ and mothers’ parenting styles and dimensions

	Authoritative				Indulgent				Authoritarian				Neglectful			
	12 years	13–14 years	15–16 years	Total	12 years	13–14 years	15–16 years	Total	12 years	13–14 years	15–16 years	Total	12 years	13–14 years	15–16 years	Total
Father																
*N*	38	114	106	258 (25.4)	95	117	42	254 (25.0)	31	146	130	307 (30.2)	31	146	130	198 (19.5)
(%)	(18.1)	(24.1)	(31.7)		(45.2)	(24.7)	(12.6)		(14.8)	(30.9)	(38.9)		(14.8)	(30.9)	(38.9)	
CR	− 2.7	− 0.9	3.3		7.6	− 0.2	− 6.4		− 5.5	0.4	4.2		− 5.5	0.4	4.2	
Warmth, *M* (SD)				74.58 (2.76)				74.77 (2.88)				58.66 (10.07)				58.93 (10.18)
Control, *M* (SD)				33.60 (4.02)				24.26 (2.93)				34.35 (4.55)				24.14 (3.47)
Mother																
*n*	46	127	113	286 (27.6)	79	113	47	239 (23.1)	38	151	109	298 (28.8)	51	91	71	213 (20.6)
(%)	(21.5)	(26.3)	(33.2)		(36.9)	(23.4)	(13.8)		(17.8)	(31.3)	(32.1)		(23.8)	(18.9)	(20.9)	
CR	− 2.2	− 0.8	2.8		5.4	0.3	− 4.9		− 4.0	1.7	1.6		1.3	− 1.2	0.2	
Warmth, *M* (SD)				76.25 (2.08)				76.29 (2.04)				63.73 (8.49)				63.76 (9.01)
Control, *M* (SD)				31.53 (4.43)				22.59 (2.59)				32.66 (5.06)				22.58 (3.01)

The analyses show that parenting style did not vary as a function of sex of adolescents [father: *χ*^2^ (3) = 3.44, *p* = 0.33, *V*_Cramer_ = .06; mother: *χ*^2^ (3) = 4.13, *p* = .25, *V*_Cramer_ = .06] but did vary as a function of adolescents’ age, with younger adolescents relatively more likely to characterise their parents as indulgent and older adolescents relatively more likely to characterise them as authoritarian [father: *χ*^2^ (6) = 92.24, *p* < 0.01, *V*_Cramer_ = .22; mother: *χ*^2^ (6) = 50.68, *p* < .01, *V*_Cramer_ = .16].

### Parenting and adolescents’ maladjustment

Descriptive analyses (Table 3) and MANOVAs (Table 4) were performed to test differences between the levels of clinical and school maladjustment among adolescents with parents reported to have high/low warmth and control and different parenting styles.

**Table 3:** Descriptive results for maladjustment by fathers’ and mothers’ parenting dimensions and styles

	Clinical maladjustment			School maladjustment		
	*M*	SD	*n*	*M*	SD	*n*
Father						
Warmth						
Low	51.46	11.61	453	54.63	9.93	453
High	46.67	10.44	447	50.40	9.75	447
Control						
Low	50.77	11.82	391	52.08	9.87	391
High	47.78	10.71	509	52.88	10.20	509
Style						
Authoritative	45.49	10.11	232	50.75	10.09	232
Indulgent	47.93	10.65	215	50.03	9.38	215
Authoritarian	49.70	10.83	277	54.66	9.96	277
Neglectful	54.23	12.26	176	54.58	9.90	176
Mother						
Warmth						
Low	51.51	11.81	456	54.95	9.70	456
High	46.59	10.16	444	50.04	9.82	444
Control						
Low	51.15	11.69	395	52.71	9.75	395
High	46.52	10.76	505	52.39	10.30	505
Style						
Authoritative	45.64	9.84	243	49.82	9.75	243
Indulgent	47.73	10.45	201	50.31	9.93	201
Authoritarian	49.15	11.30	262	54.77	10.25	262
Neglectful	54.70	11.77	194	55.21	8.92	194

**Table 4:** Results of multivariate and univariate analyses of variance for maladjustment

	*F*	DF	*p*
Father’ dimensions			
Warmth	34.74	2, 924	< .001
Clinical maladjustment	52.18	1,925	< .001
School maladjustment	46.51	1, 925	< .001
Control	15.98	2, 924	< .001
Clinical maladjustment	24.00	1, 925	< .001
School maladjustment	0.26	1, 925	.612
Warmth × control	0.98	2, 924	.373
Mother’ dimensions			
Warmth	41.71	2, 945	< .001
Clinical maladjustment	51.96	1, 946	< .001
School maladjustment	64.94	1, 946	< .001
Control	18.34	2, 945	< .001
Clinical maladjustment	32.94	1, 946	< .001
School maladjustment	0.30	1, 946	.582
Warmth × control	2.33	2, 945	.098
Styles			
Father	4.06	6, 1768	< .001
Clinical maladjustment	5.66	3, 884	.001
School maladjustment	3.60	3, 884	.013
Mother	8.00	6, 1768	< .001
Clinical maladjustment	14.98	3, 884	< .001
School maladjustment	5.05	3, 884	.002
Father × mother	1.81	6, 1768	.020
Clinical maladjustment	2.49	9, 884	.008
School maladjustment	1.39	9, 884	.186

#### Parents’ warmth and control and adolescents’ maladjustment

The interaction between fathers’ warmth and control was not statistically significant; however, the main effects of fathers’ warmth and control were significant. Follow-up ANOVA revealed significant differences in clinical maladjustment depending on the father’s warmth and control. Adolescents whose fathers had low levels of warmth scored more highly in clinical maladjustment than those with fathers presenting high levels of warmth [*HSD*_Bonferroni_ = 5.24, *p* < .001, *g´* = 0.43]. Similarly, students whose fathers had low levels of control had higher levels of clinical maladjustment than those whose fathers had higher levels of control [*HSD*_Bonferroni_ = 3.55, *p* < .001, *g´* = 0.27]. ANOVAs also indicated that there were significant differences in school maladjustment depending on the father’s warmth but there were no such differences with respect to control. Adolescents whose fathers presented low levels of warmth scored higher in school maladjustment than those whose fathers presented high levels of warmth [*HSD*_Bonferroni_ = 4.5, *p* < .001, *g´* = 0.43].

As for the mothers, the results were similar: the main effects of mothers’ warmth and control were significant and the interaction between mothers’ warmth and control was not significant. In this case, too, follow-up ANOVAs revealed significant differences in clinical maladjustment depending on the mother’s warmth and control and differences in school maladjustment depending only the mother’s warmth. Adolescents whose mothers presented low levels of warmth had higher levels of clinical maladjustment [*HSD*_Bonferroni_ = 5.15, *p* < .001, *g´* = 0.45] and school maladjustment [*HSD*_Bonferroni_ = 5.13, *p* < .001, *g´* = 0.50]. Higher levels of clinical maladjustment were also found among the adolescents whose mothers had low levels of control compared to those reporting high levels of maternal control [*HSD*_Bonferroni_ = 4.10, *p* < .001, *g´* = 0.41].

#### Parenting styles and adolescents’ maladjustment

MANOVA was performed with the scores for school and clinical maladjustment as dependent variables and with the mothers’ or fathers’ parenting styles as the independent variable. The interaction between the mothers’ and fathers’ parenting styles was significant but only for clinical maladjustment. The main effects of the mothers’ and fathers’ parenting styles were found to be significant in all variables.

**Fig. 2:**
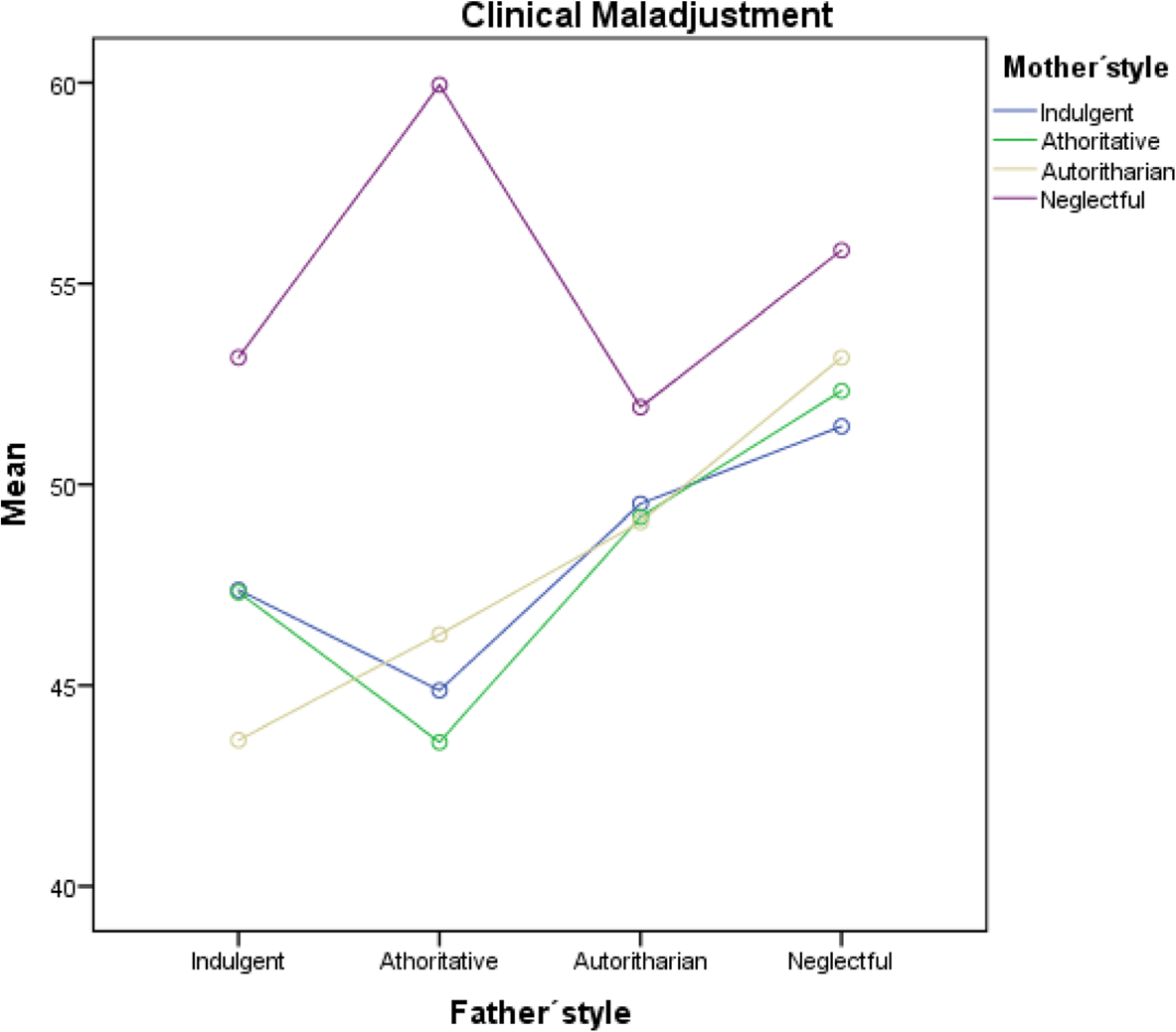
Adolescents’ clinical maladjustment by parent’ styles

Follow-up ANOVA and post hoc HSD Tukey with Bonferroni test (see Fig. 2 and Table 5) for the significant interaction showed that students with neglectful mothers and authoritative fathers presented the highest levels of clinical maladjustment, followed by those with other combinations of neglectful styles, authoritarian, indulgent and finally authoritative styles. No statistically significant differences were found between combinations of fathers’ and mothers’ indulgent and authoritative styles, or between neglectful and authoritarian combinations.

**Table 5:** Descriptives and differences between sub-groups in clinical maladjustment

Father’style	Mother’style	*M*	SD	*N*	Sub-group			
					1	2	3	4
Authoritative	Authoritative	43.49	8.67	155	*			
Indulgent	Authoritarian	43.64	10.97	14	*			
Authoritative	Authoritarian	46.27	9.64	37	*	*		
Indulgent	Authoritative	47.32	10.04	37	*	*		
Indulgent	Indulgent	47.33	10.53	135	*	*		
Authoritarian	Authoritarian	49.07	11.10	162	*	*	*	
Authoritarian	Neglectful	51.93	9.99	56		*	*	
Indulgent	Neglectful	53.17	10.42	30		*	*	*
Neglectful	Neglectful	55.83	12.81	89			*	*
Authoritative	Neglectful	59.95	11.84	19				*
*p*					.377	.117	.135	.132

Note: *belongs to the subgroup

Follow-up ANOVA indicated that the effects of both mothers’ and fathers’ parenting styles were significant for school maladjustment. The results for mothers’ and fathers’ parenting styles were in the same line. The post hoc with Bonferroni correction tests revealed statistically significant differences between neglectful/authoritarian and indulgent/authoritative families; the students from neglectful and authoritarian families presented highest levels of school maladjustment [father: DHS_neglectful vs indulgent_ = 4.50, *p* < .001, *g´* = 0.44, DHS_neglectful vs authoritative_ = 3.83, *p* = .001, *g´* = 0.38, DHS_authoritarian vs indulgent_ = 4.63, *p* < .001, *g´* = 0.41, DHS_authoritarian vs authoritative_ = 3.90, *p* < .001, *g´* = 0.32; mother: DHS_neglectful vs indulgent_ = 4.90, *p* < .001, *g´* = 0.52, DHS_neglectful vs authoritative_ = 5.39, *p* < .001, *g´* = 0.57, DHS_authoritarian vs indulgent_ = 4.46, *p* < .001, *g´* = 0.44, DHS_authoritarian vs authoritative_ = 4.95, *p* < .001, *g´* = 0.49]. No significant differences were found between neglectful and authoritarian families [father: DHS = − 0.08, *p* = .999; mother: DHS = 0.44, *p* = .964], or between indulgent and authoritative families [father: DHS = − 0.73, *p* = .859; mother: DHS = 0.40, *p* = .952], in this respect.

## Discussion

This study was conducted to analyse the relationships between adolescents’ adjustment (clinical and school) and parenting styles using two dimension (warmth and control) and four typologies (authoritarian, authoritative, negligent and indulgent), with a sample of Spanish adolescent students.

Severity levels of clinical and school maladjustment were explored, and approximately 5% of the adolescents were found to have clinically significant adjustment problems, a finding that is in line with the previous studies (Donaldson et al., 2014; Merikangas et al., 2010). One of the strong points of the BASC-S3 is that clinically significant and at-risk results are obtained, so that early intervention can be made to help adolescents who are at risk of clinical or school maladjustment. The results of the present study highlight the need to take into account adolescents who may be at risk of maladjustment, because the percentages obtained are far from negligible (10.5% for clinical maladjustment and 18.8% for school maladjustment). Moreover, age but not sex was relevant to severity levels for adolescents’ adjustment problems. Younger adolescents were more prone than others to suffer clinically significant clinical maladjustment and middle adolescents to suffer risk for school maladjustment and clinically significant school maladjustment. In contrast, when analysing the scores, statistically significant effect of the sex and age group interaction on the clinical maladjustment and main effect of sex—with low effect size—and age on school maladjustment were found. Thus, as girls become older, they score higher in clinical maladjustment, but boys present the opposite trend. Many previous studies have reported higher levels of emotional problems among adolescent girls than boys (Compas, Connor-Smith, & Jaser, 2004; Waite & Creswell, 2014), probably because they are more sensitive to the typical changes of adolescence as these changes come over (and possibly, also, due to greater social pressures). On the contrary, boys are more prone to school maladjustment, which is in line with previous findings (Holden, 2002; Lam et al., 2012). Although externalising problems have been understood as stable over the course of development (Li et al., 2017), the present study found the 13–14 age group as the one with highest levels of school maladjustment, maybe because it is also related with a level change in Spanish Education (from Primary to Secondary Education).

The next aim of our study was to analyse the parenting dimensions and styles of fathers and mothers separately, according to their adolescent children’s perceptions. One of the main conclusions drawn is that, as concluded by Sheeber et al. (2007), there were no significant differences between adolescents’ perceptions of their mothers’ and fathers’ dimensions and parenting styles, which contradicts the results of other studies (Hoeve et al., 2009; McKinney et al., 2008).

To obtain more detailed information about parenting, we analysed the two main dimensions of parenting (warmth and control). Hypothesis 1 was confirmed, with our results showing that adolescent maladjustment was most likely to occur when adolescents perceived low warmth and acceptance from their parents. The results showed, too, that only fathers’ and mothers’ warmth was significantly associated with school maladjustment (parental control was non-significant): thus, low warmth was related to higher levels of school maladjustment. These results coincide with previous findings about parenting styles, reinforcing the idea that no significant differences arise between indulgent and authoritative families as regards school maladjustment; what really matters is the parents’ level of warmth. On the other hand, the results for clinical maladjustment argue in favour of an authoritative parenting style, with higher levels of warmth and control being related to lower levels of clinical maladjustment. In this case, therefore, parental control is an important factor, and so hypothesis 2 (that adolescent maladjustment is more likely to occur when adolescents perceive low control and supervision from their parents) was partially confirmed, being true for clinical but not for school maladjustment.

The analysis of the relationship between parenting styles and clinical maladjustment revealed that the interaction between the mothers’ and fathers’ parenting styles was significant; the highest level of maladjustment was presented for the students with neglectful mothers and authoritative fathers, followed by other combinations for neglectful mothers. In the case of school maladjustment, revealed similar results for mothers and fathers, but with higher effect sizes associated in the case of different mothers’ parenting styles. Adolescents’ perceptions of neglectful or authoritarian fathers and mothers is related to higher levels of maladjustment, while perceptions of authoritative or indulgent fathers and mothers is related to lower levels of maladjustment. No significant differences were found between indulgent and authoritative families or between neglectful or authoritarian, which indicates that these two parenting styles have similar effects on adolescents, as suggested by previous studies conducted in Spain (Gámez-Guadix et al., 2012; García & Gracia, 2009; López-Romero et al., 2012). These results corroborate our hypothesis 3 and highlight the positive effect of high levels of parental warmth (present in indulgent and authoritative families).

It seems that in the Spanish culture, a high level of affection (present in both the authoritative and the indulgent styles) is considered very important, and that these styles are distinguished by the level of imposition or coercive control exerted (Musitu & García, 2004). By contrast, other authors argue that superior outcomes are achieved by the authoritative style of parenting, in all cultures (see, for example, Sorkhabi, 2005; Steinberg et al., 2006). In view of these conflicting outlooks, further study is needed of the relationship between children’s adjustment/maladjustment and parenting styles, in different cultural contexts.

## Conclusions

In summary, these results are not as conclusive as other studies conducted in Spain, which have argued that the indulgent style of parenting is “optimum” (Esteve, 2005; García & Gracia, 2009, 2010). Following the ideas of Steinberg (2001), we find that authoritative families seem to be especially important for adolescents’ emotional well-being; besides the importance of the positive expression of affect and support provided by such parents, it is also important to set limits and establish structures for children to facilitate their development of self-regulatory skills.

Finally, the limits of the study must be taken into account. Its cross-sectional design and reliance on self-reporting by adolescents mean that the results obtained should be interpreted with caution. Moreover, causal conclusions cannot be drawn. Although studies show that adolescents’ perceptions of their mothers’ and fathers’ parenting styles yield reliable results (Baumrind, 1991; Lamborn et al., 1991; Steinberg, Lamborn, Darling, Mounts, & Dornbusch, 1994), it would nevertheless be interesting to compare these results with the views of the parents concerned. Lastly, a better understanding is needed of the relationship between parenting and adolescents’ levels of personal adjustment in different cultures, to identify the aspects of parental socialisation that are determinant factors in children’s psychosocial development.
